# The effect of tart cherry juice (TCJ) supplementation on exercise-induced muscle damage (EIMD) in an athletic population

**DOI:** 10.1097/MS9.0000000000002914

**Published:** 2025-01-21

**Authors:** Elaheh Dehghani, Mohammad Beba, Khashayar Danandeh, Amirhossein Memari, Mohamad Javad Ershadmanesh, Pegah Rasoulian, Arshiya Danandeh, Kurosh Djafarian

**Affiliations:** a Department of Clinical Nutrition, School of Nutrition and Dietetics, Tehran University of Medical Sciences (TUMS), Tehran, Iran; b Sports Medicine Research Center, Neuroscience Institute, Tehran University of Medical Sciences, Tehran, Iran; c Medicine Faculty, Shahid Beheshti University of Medical Sciences (SBMU), Tehran, Iran

**Keywords:** exercise-induced muscle damage, inflammatory biomarkers, maximal voluntary isometric contraction, meta-analysis, muscle function, tart cherry juice

## Abstract

**Introduction:**

This systematic review and meta-analysis quantified the effect of tart cherry juice (TCJ) supplementation on exercise-induced muscle damage (EIMD). Evidence supported TCJ’s beneficial effects on muscular function and inflammatory biomarkers interleukin (IL)-6 and IL-8.

**Method:**

PubMed, Scopus, and Web of Science were searched up to January 2024. Risk of bias was assessed using RevMan® software. Mean differences and 95% confidence intervals (CIs) of maximal voluntary isometric contraction (MVIC), inflammatory markers, creatine kinase (CK), and visual analog scale (VAS) score were pooled using fixed- or random-effect models. Heterogeneity was assessed using Chi-square or *I*^2^ statistics.

**Results:**

Ten trials were included in the analysis. TCJ supplementation significantly improved MVIC [weighted mean difference (WMD) = 9.13%, 95% CI (6.42–11.84), *I*^2^ = 62.3%] and decreased IL-6 [WMD = −0.4 pg/ml, 95% CI (−0.68 to −0.11), *I*^2^ = 62.2%] and IL-8 [WMD = −0.3 pg/ml, 95% CI (−0.6 to −0.0), *I*^2^ = 46.3%]. No significant changes were found in CK, C-reactive protein, IL-1β, tumor necrosis factor-alpha, or VAS score. Dose–response analysis revealed a significant non-linear association between daily TCJ dose and MVIC effect size.

**Conclusion:**

TCJ supplementation may improve muscle function and some inflammatory biomarkers in EIMD. Further high-quality studies with larger sample sizes are needed to determine TCJ’s long-term effects.

## Introduction

The epidemic of physical inactivity is one of the largest public health issues in the twenty-first century^[1–3]^. Participation in athletic events is a strategy to promote an active lifestyle, which has numerous proven benefits^[4–8]^. On the other hand, sports-related injuries are considered an undesirable aspect of this process and may prevent engagement in physical activity^[9]^. Sports-related injuries should, therefore, be avoided because they can interfere with efforts to promote physical activity and come at a high cost to society^[6]^. Exercise-induced muscle damage (EIMD) is brought on by hard or sustained (or both of them together) exercise, particularly following eccentric exercises^[10]^, and is thought to occur in two stages^[11]^. The initial phase of muscle injury caused by arduous exercise comprises a mechanical disruption of sarcomeres and oxidative stress resulting from a rise in reactive oxygen species (ROS) production^[12]^. An inflammatory reaction caused by the primary muscle damage results in secondary muscle damage, which may postpone a full recovery of muscle function^[13–17]^. Numerous therapies have been used in this regard, such as cold water immersion, compression clothing, non-steroidal anti-inflammatory drugs, and dietary supplements having antioxidant or anti-inflammatory properties^[11,18]^. When a quick recovery is required, such as during multi-day events, these interventions may be helpful^[19]^. Dietary supplements with anti-inflammatory and antioxidant properties are considered to decrease inflammation and fasten the healing process^[20]^. In sports, tart cherry (TC) supplements are frequently utilized for reasons other than performance improvement^[21]^; however, they are also used to alleviate EIMD symptoms and fasten recovery, since TC supplements are abundant in phenolic components, which may have anti-inflammatory and antioxidant effects^[15,20,22]^. These substances’ antioxidant and anti-inflammatory characteristics may assist athletes in recuperating from the oxidative stress brought on by free radicals, as well as reduce inflammation and EIMD^[23–25]^. The polyphenolic compounds have hydroxyl groups attached to ring structures that give them antioxidant properties^[26]^. These compounds can act as chelating agents for iron or copper or as free radical scavengers to protect cells from oxidative damage^[24,25]^. Additionally, polyphenolic compounds can indirectly promote the expression of genes for antioxidant enzymes like superoxide dismutase or glutathione peroxidase and limit the activity of the ROS-producing enzymes^[24]^. In addition, the meta-analysis by Gao and Chilibeck highlights the efficacy of TC concentrate in improving endurance exercise performance through mechanisms such as enhanced oxygen delivery and reduced oxidative stress^[27]^.

Results from clinical trials investigating the effect of TC supplementation on EIMD are conflicting. Some clinical trials have shown a significant reducing effect of tart cherry juice (TCJ) supplementation on some markers of EIMD^[27–35]^, while others did not find any significant effect^[15,36]^. Therefore, we believe that the existence of a systematic review and meta-analysis summarizing all available findings in this area can clarify the overall effect of TCJ supplementation on EIMD. Therefore, the current systematic review and meta-analysis were conducted to systematically review the current evidence on the effect of TCJ on EIMD in the athletic population in a dose–response manner. To the best of our knowledge, no previous study investigated the effect of TCJ supplementation on different aspects of EIMD, which shows the novelty of the present study.

## Methods

The preferred reporting items for systematic reviews and meta-analyses (PRISMA) statement was used for conducting the current investigation^[37]^.

### Search strategy

A thorough search was done by two authors (E.D. and M.B.) in PubMed, Scopus, and Web of Science up to January 2024 to identify the eligible papers. The following search keywords were used together: [(tart cherry juice, TC Juice, Montmorency, tart cherry, Montmorency cherry) And (sports injury, muscle soreness, muscle pain, muscle damage, athletic injury, sports injury, athletic injury, muscle damage, delayed onset muscle soreness) And (random allocation, single-blind method, double-blind method, crossover studies, clinical trials as topic, randomized controlled trial (RCT), intervention studies, controlled trial, randomized, random, randomly, placebo, assignment, trial, crossover procedure, equivalence trial)]. There were no language or publishing period constraints, and the references list of the relevant papers was also checked to make sure no linked publications were missed. To manage and accelerate the review process, all studies were moved to EndNote (version X9.3.3).

### Study selection

Studies were chosen using the population-intervention-comparison-outcome-study design (PICOS) and were considered if they satisfied the following requirements: (1) randomized controlled clinical trials; (2) research on physically active adults (≥18 years); (3) original studies looking at a short- or long-term TCJ supplementation; (4) lack of significant methodological problems (e.g., absence of placebo or control group, participant not blinded, and improper statistical analysis procedures); and (5) studies that provided the necessary data to determine the effect sizes, such as the means and standard deviations (SDs) of creatine kinase (CK), C-reactive protein (CRP), interleukin (IL)-1B, IL-6, IL-8, tumor necrosis factor-alpha (TNF-α), maximal voluntary isometric contraction (MVIC), and visual analog scale (VAS) for both intervention and control groups. Studies were excluded if they (1) employed TCJ supplementation in combination with other nutrients or dietary supplements; (2) were ecological studies, cross-sectional studies, case-control studies, systematic reviews, or meta-analyses; or (3) were trials without a control group. After the initial search, the EndNote software was used to screen all recorded items retrieved from the electronic search (version X9.3.3). Study titles and abstracts were chosen by the inclusion criteria after being examined separately by two reviewers (E.D. and M.B.). Studies that met the eligibility criteria during the title and abstract screening were chosen for full-text review.

### Data extraction

Data extraction was performed by two independent researchers (E.D. and M.B.). The following information was collected: the first author’s name, the year of publication, the subjects’ characteristics [mean age, height, weight, body mass index (BMI), and gender], the study’s design, the number of participants (in the control and intervention groups), the type of exercise, the dosage of TC supplementation (volume/day), the anthocyanin content of supplements, duration of intervention, and the mean changes and their corresponding SDs of CK, CRP, IL-1B, IL-6, IL-8, TNF-α, MVIC, and VAS throughout the trials for the intervention and control groups.

### Data analysis

The pooled effect size was expressed as weighted mean difference (WMD) and its corresponding 95% confidence interval (CI) for each parameter in the present meta-analysis. The heterogeneity of the included studies was examined by χ^2^ tests and the degree of heterogeneity was estimated using the *I*^2^ statistic. A fixed-effect model (when *I*^2^ was below 50%) or random-effect model (when *I*^2^ was above 50%) was used for the meta-analysis. Subgroup analysis was performed based on the type of drink, country, study design, gender, BMI, company, and type of exercise to discern the potential source(s) of heterogeneity. Since the type of TCJ affects its antioxidant content, we decided to consider the company as a potential source of heterogeneity. Potential publication bias was explored by using Egger’s line regression test (Egger’s test). All analyses were performed by STATA software (version 12.0), and *P* < 0.05 was considered statistically significant. In instances where the standard error of mean (SEM) was reported, it was converted to SDs through the following formula: SD = SEM ×√*N* (*N* is the number of participants in each group). In articles that reported data in graphical figures, data extraction was performed using a Web plot digitizer. Sensitivity and dose–response analyses were performed using “metaninf” and “fracpoly” commands, respectively, by STATA software (version 12.0).

## Results

### Description of studies

**Figure 1.**
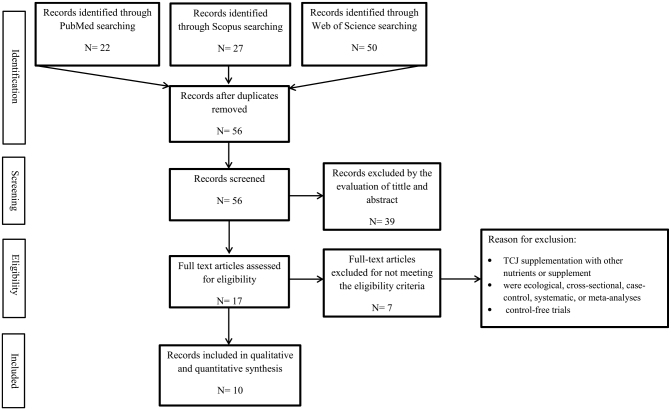
Flow diagram of the selection process.

A total of 109 articles were found in the original search; however, 27 were dropped due to duplication. Based on their irrelevant titles and abstracts, 39 further papers were eliminated. Seven studies were further removed after a full-text review of 33 possibly relevant articles because they did not report our intended results Finally, 10 RCT-designed papers were picked for the systematic review and meta-analysis. Fig. 1 shows the flowchart for the selection procedure.

### Study characteristics

Table 1 shows the characteristics of the 10 RCTs in the current systematic review and meta-analysis. These RCTs were published between 2009 and 2022 and were from Australia^[35]^, the UK^[15,28–32,34]^, and the USA^[33,36]^. Six studies were exclusively performed on male subjects^[27–30,34,36]^ and four studies were conducted on both genders^[32–34,36]^. The sample size of included RCTs varied from 16 to 51 participants, resulting in a total sample size of 212 individuals. The mean age of participants was between 23 and 37 years. The dosage of TC supplements varied from 60 to 1200 ml/day, and the duration of intervention ranged from 7 to 12 days across selected RCTs. Most studies employed parallel design^[15,28–30,32–34,36]^, while two studies used a crossover design^[31,35]^. Three studies measured IL-1B^[28–30]^ and IL-8^[28–30]^, whereas TNF-α was measured in four^[28,30,35]^ studies. Additionally, five trials^[28,30,32,35]^ investigated IL-6, and CRP was measured in six^[28–31,34,35]^, MIVC in seven^[15,28,30–32,34,35]^, and CK in eight^[15,28–32,34,35]^ investigations.

**Table 1:** Characteristics of included studies

Author,year	Country	Study design	Type of exercise	Groups	Age	Height (cm)	Weight (kg)	BMI	Volume/day (ml)	Anthocyanin (mg/L)	Duration (day)	Article used for reporting polyphenol content	Outcomes	Results
Howatson, 2009	UK	Parallel	Marathon run	TCJ:10 PLA:10 (F/M) Trained subjects	37.5	176	73.35	23.67	480	80	8	Connolly *et al*.	CK, IL-6, MIVC, VAS	↑Recovery of strength, MIVC, total antioxidant capacity, ↓lipid peroxidation, IL-6, CRP
Bowtell, 2010	UK	Crossover	Strenuous exercise	TCC:10 PLA:10 (M) Trained subjects	27.8	176	81.3	26.24	60	273.5	10	Study estimates	CK, CRP, MIVC, PPT	↑Recovery of isometric muscle strength, ↓oxidative stress, and inflammation, CRP
Kuehl, 2010	USA	Parallel	Running	TCJ:26 PLA:25 (F/M) Trained subjects	35.8	-	-	25.6	710	80	8	Connolly *et al*.	VAS	↓ Symptoms of EIMD
Bell, 2014	UK	Parallel	Cycling	TCJ:8 PLA:8 (M) Trained subjects	30	181.1	76.5	23.35	260	273.5	7	Bowtell *et al*.	CK, CRP, IL-1B, IL-6, IL-8, TNF-α, LOOH	Attenuated oxidative stress and inflammation, ↓ LOOH, CRP, IL-6
Bell, 2015	UK	Parallel	Cycling	TCJ:8 PLA:8 (M) Trained subjects	30	181.1	76.5	23.35	260	273.5	8	Bowtell *et al*.	CK, CRP, IL-1B, IL-6, IL-8, TNF-α, MIVC, VAS, LOOH	Attenuation of inflammation, recovery, MIVC IL-6, CRP
Beals, 2016	USA	Parallel	Eccentric exercise	TCJ:15 PLA:14 (F/M) Trained subjects	25.25	173.1	70.05	23.30	1200	128	12	Study estimates	ROM, VAS	No significant differences between groups
Bell, 2016	UK	Parallel	Sprint	TCJ:8 PLA:8 (M) Trained subjects	25	180.8	81.9	25.27	260	273.5	8	Keane *et al*.	CK, CRP, IL-1B, IL-6, IL-8, TNF-α, MIVC, CMJ, VAS, LOOH	↑Recovery, benefit for sprinting or high-intensity directional changes, ↑MIVC, CMJ ↓IL-6
Lamb, 2019	UK	Parallel	Eccentric exercise	TCJ:12 PLA:12 (M) Trained subjects	24	-	-	25.60	500	273.5	9	Study estimates	CK, MIVC, ROM, VAS	No significant differences between groups
Quinlan, 2019	UK	Parallel	Football/hockey	TCJ:10 PLA:10 (F/M) Trained subjects	26	175.4	70.2	22.92	200	548	8	Did not describe the details	CK, CRP, MIVC, CMJ, VAS	↑ Recovery, CMJ, MVIC, antioxidant and inflammation, ↓ CK
Wangdi, 2022	Australia	Crossover	Eccentric exercise	TCC:10 PLA:10 (M) Trained subjects	23.4	178.4	78	24.61	60	273.5	10	Study estimates	CK, CRP, IL-6, TNF-α, MIVC	↓Recovery from EIMD, antioxidant

AC, anthocyanin content; CK, creatine kinase; CMJ, countermovement jump; CRP, C-reactive protein; EIMD, exercise-induced muscle damage; F, female; IL-1B, interleukin-1B; IL-6, interleukin-6; IL-8, interleukin-8; LOOH, lipid hydroperoxides; M, male; MIVC, maximal isometric voluntary contraction; PLA, placebo; PPT, pressure pain threshold; RCT, randomized controlled trial; ROM, range of motion; TCC, tart cherry concentrate; TCJ, tart cherry juice; TNF-α, tumor necrosis factor-α; UK, united kingdom; USA, united states of America; VAS, visual analog scale; ↓, decrease; ↑, increase.

### Risk of bias

**Figure 2:**
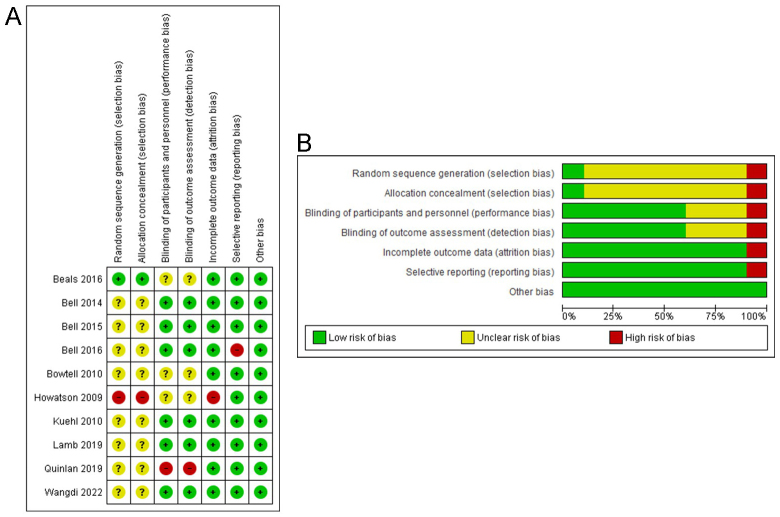
(A) Risk of bias summary: review authors’ judgments about each risk of bias item for each included study. (B) Risk of bias graph: review authors’ judgments about each risk of bias item presented as percentages across all included studies.

The assessment of the risk of bias was performed using RevMan® software (version 5.3), and the final result is presented in Fig. 2A. The review of authors’ judgments about each risk of bias item as a percentage across all included studies is also presented in Fig. 2B.

### Meta-analysis

#### Effect of TCJ supplementation on muscle function

**Figure 3.**
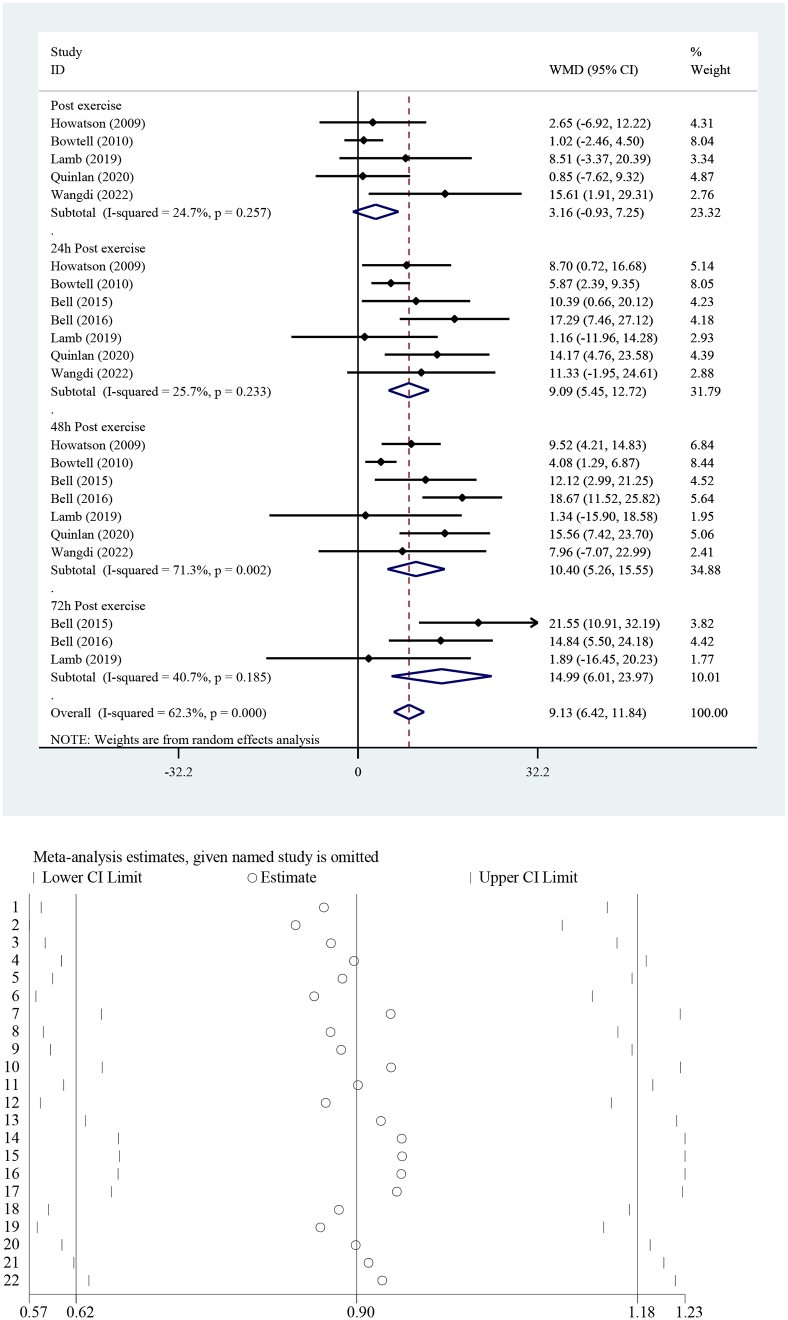
Forest plot of the effect of tart cherry juice supplementation on MVIC in the intervention group compared to the placebo. MVIC, maximal isometric voluntary contraction.

The effect of TCJ supplementation on MVIC is shown in Fig. 3. The pooled analysis showed that TCJ supplementation increased MVIC significantly [WMD = 9.13% from baseline, 95% CI (6.42–11.84), *I*^2^ = 62.3%] compared to the placebo group (*P* < 0.001). Due to the high level of heterogeneity, subgroup analysis was performed based on the type of drink, country, study design, gender, BMI, company, and type of exercise to discern the potential sources of heterogeneity, which is shown in Supplemental Table 1, http://links.lww.com/MS9/A671.

#### Effect of TCJ supplementation on inflammatory cytokines

##### IL-6

**Figure 4.**
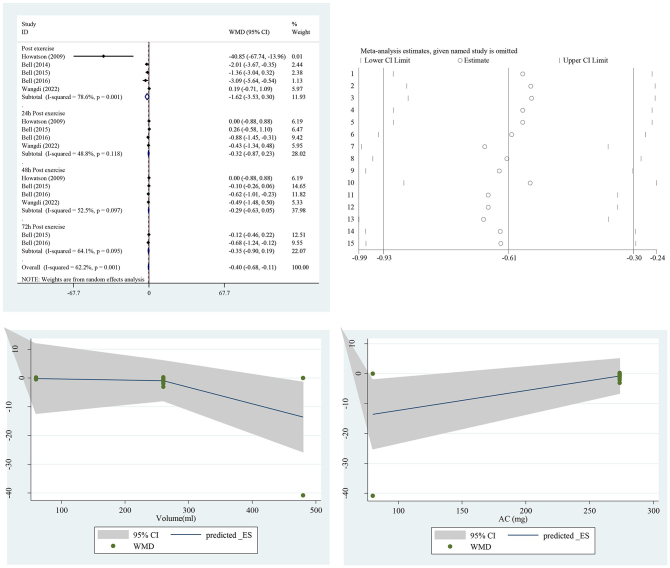
Forest plot of the effect of tart cherry juice supplementation on IL-6 in the intervention group compared to the placebo. IL, interleukin. (dose 1: p = 0.07 and dose 2: p = 0.07). (dose 1: p = 0.052 and dose 2: p = 0.052).

The effect of TCJ supplementation on IL-6 is presented in Fig. 4. The pooled analysis showed that TCJ supplementation significantly reduced IL-6 levels in the intervention group [WMD = −0.40 pg/ml, 95% CI (−0.68 to −0.11), *I*^2^ = 62.2%] compared to the placebo group (*P* = 0.006). Due to the high level of heterogeneity, subgroup analysis was performed based on the type of drink, country, study design, gender, BMI, company, and type of exercise to discern the potential sources of heterogeneity, which is presented in Supplemental Table 2, http://links.lww.com/MS9/A672.

##### IL-8

**Fig. 5.**
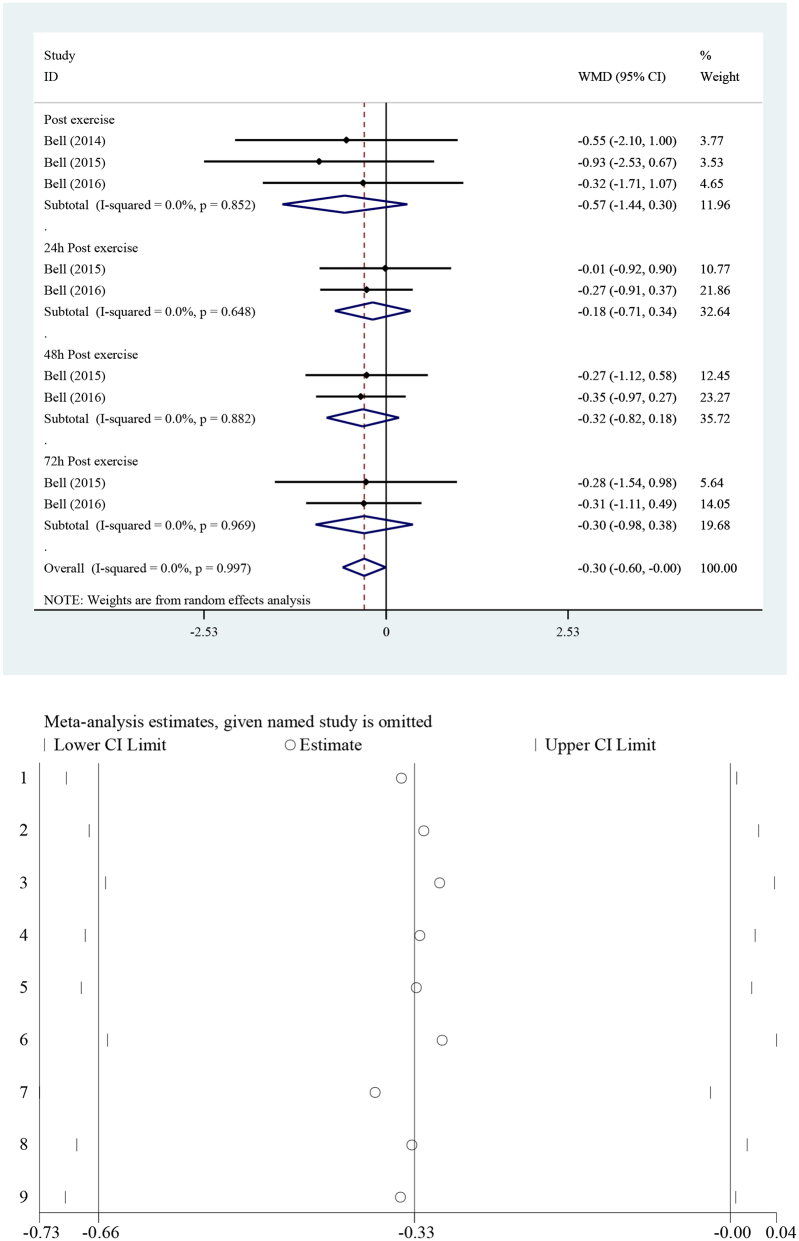
Forest plot of the effect of tart cherry juice supplementation on IL-8 in the intervention group compared to the placebo. IL, interleukin. Dose-response = 1.

The effect of TCJ supplementation on IL-8 is shown in Fig. 5. The pooled analysis showed that TCJ supplementation significantly reduced IL-8 levels in the intervention group [WMD = −0.30 pg/ml, 95% CI (−0.60 to −0.00), *I*^2^ = 0.0%] compared to the placebo group (*P* = 0.04).

##### IL-1β

The effect of TCJ supplementation on IL-1β is shown in Supplemental Fig. 1, http://links.lww.com/MS9/A673. The pooled analysis showed that TCJ supplementation was not able to significantly reduce IL-1β levels in the intervention group [WMD = −0.00 pg/ml, 95% CI (−0.03 to 0.02), *I*^2^ = 19.1%] compared to the placebo group (*P* = 0.73).

##### TNF-α

The effect of TCJ supplementation on TNF-α is shown in Supplemental Fig. 2, http://links.lww.com/MS9/A674. The pooled analysis showed that TCJ supplementation was not able to significantly reduce TNF-α levels [WMD = −0.11 pg/ml, 95% CI (−0.25 to 0.02), *I*^2^ = 6.2%] compared to the placebo group (*P* = 0.09).

##### CRP

The effect of TCJ supplementation on CRP is presented in Supplemental Fig. 3, http://links.lww.com/MS9/A675. The pooled analysis revealed that TCJ supplementation was not able to significantly reduce CRP levels in the intervention group [WMD = −0.08 pg/ml, 95% CI (−0.26 to 0.10), *I*^2^ = 0%] compared to the placebo group (*P* = 0.39).

#### Effect of TCJ supplementation on muscle damage

The effect of TCJ supplementation on CK levels is shown in Supplemental Fig. 4, http://links.lww.com/MS9/A676. The pooled analysis indicated that TCJ supplementation increased CK level significantly [WMD = 45.88 IU/L, 95% CI (3.64–88.13), *I*^2^ = 35.6%] in the intervention group compared to the placebo group (*P* = 0.03).

#### Effect of TCJ supplementation on muscle soreness

The effect of TCJ supplementation on VAS is presented in Supplemental Fig. 5, http://links.lww.com/MS9/A677. The pooled analysis showed that TCJ supplementation was not able to significantly decrease VAS [WMD = −2.81, 95% CI (−9.46 to 3.85), *I*^2^ = 66.1%] compared to the placebo group (*P* < 0.001).

### Sensitivity analysis and publication bias

Results of sensitivity analysis indicated that our final result was not significantly influenced by a specific study. In other words, the included studies possessed an almost equal weight. Funnel plots of CK, CRP, IL-1β, IL-6, IL-8, TNF-α, MVIC, and VAS are shown in Supplemental Figures 6–13, http://links.lww.com/MS9/A678, http://links.lww.com/MS9/A679, http://links.lww.com/MS9/A680, http://links.lww.com/MS9/A681, http://links.lww.com/MS9/A682, http://links.lww.com/MS9/A683, http://links.lww.com/MS9/A684, http://links.lww.com/MS9/A685, respectively. Begg’s test showed no evidence of publication bias for CK (*P* = 0.65), CRP (*P* = 0.24), IL-1β (*P* = 0.21), IL-8 (*P* = 0.14), and MVIC (*P* = 0.88). However, significant publication bias was observed for IL-6 (*P* = 0.023), TNF-α (*P* = 0.006), and VAS (*P* = 0.025).

### Dose–response analysis

We tested for a non-linear association between TCJ supplementation and IL-6, IL-8, and MVIC to discern the probable association between the daily dose of TCJ supplementation and the final effect size. Dose 1 refers to the minimum effective daily dose of 60 ml, while dose 2 represents a higher daily dose of 260 ml. Results of the dose–response analysis revealed that TCJ supplementation failed to decrease IL-6 and IL-8 (*P* > 0.05) but did increase MVIC levels (dose 1: *P* = 0.005 and dose 2: *P* = 0.004) in a non-linear fashion. According to the present study, the optimal dose for increasing MVIC levels was 260 ml/day. Since different TCJs have different contents of anthocyanin, we decided to test further for a non-linear association between anthocyanin levels (mg/day) to find out if the anthocyanin level of the juice is responsible for this association. Results of the dose–response analysis on anthocyanin contents of TCJ revealed that this compound failed to increase MVIC levels (*P* > 0.05). The results of the dose–response analysis are shown in Supplemental Figures 14, http://links.lww.com/MS9/A686, and 15, http://links.lww.com/MS9/A687.

## Discussion

In the present systematic review and meta-analysis, we summarized published evidence from 10 clinical trials that investigated the effect of TCJ supplementation on markers of muscle function (MVIC), inflammation (CRP, IL-1B, IL-6, IL-8, TNF-α), muscle damage (CK), and muscle soreness (VAS). After TCJ supplementation a significant improvement was seen in MVIC (9.13% from baseline). Maximum voluntary isometric contraction or MVIC is a concise indicator of muscle strength^[38,39]^, and our study indicated that TCJ supplementation can significantly improve MVIC in the intervention group compared to the placebo group. Additionally, significant but partial reductions were observed in some inflammatory cytokines including IL-6 (−0.4 pg/ml) and IL-8 (−0.3 pg/ml) following TCJ supplementation. Notably, TCJ supplementation resulted in an increase in CK levels by 45.88 IU/L, contrary to expectations. The increase in CK levels suggests a complex physiological reaction where, rather than reducing muscle damage, TCJ may stimulate muscle repair mechanisms. Typically, CK elevation is considered an indicator of muscle injury; however, emerging research suggests its role in signaling pathways involved in muscle repair. Nevertheless, there is debate about whether or not it accurately reflects muscle damage caused by varying levels and intensities of physical activity. But for the muscle regeneration process to begin, CK levels must be reduced^[40]^. Several variables could contribute to the increase in CK levels after TCJ administration. There is a potential for the physiological reaction to TCJ to have a beneficial impact on several indicators, which might potentially result in an indirect stimulation of CK release through certain metabolic or biochemical interactions. On the other hand, the observed increase in levels could perhaps be a transient reaction linked to the body’s adjustment to the intake of supplements, with the possibility of eventually resulting in favorable long-term effects. The lack of significant effects on CK, CRP, IL-1β, TNF-α, and VAS may stem from variability in participant characteristics, exercise protocols, and intervention durations, as well as small sample sizes limiting statistical power. The unexpected increase in CK levels might indicate a physiological response related to muscle repair rather than damage, highlighting the need for further research with larger, standardized trials to clarify TCJ’s mechanisms and effects on EIMD. Another factor to consider is the quantity and duration of TCJ supplementation, as varying treatment plans may result in differing impacts on CK levels. Furthermore, it is important to consider that the observed elevation in CK levels following TCJ administration may be influenced by several individual factors, such as genetic predisposition, dietary choices, or lifestyle habits^[41]^. Additional investigation is required to dig deeper into the fundamental mechanisms and probable rationales for the unforeseen increase in CK levels after TCJ consumption. This phenomenon may offer useful insights for researchers and professionals operating within the realm of sports science and nutritional practices. Therefore, we can conclude that TCJ supplementation is not a good strategy to reduce CK levels as an indirect marker of muscle damage.

TCJ supplementation was also not able to reduce other markers of inflammation including CRP (−0.08 pg/ml), IL-1β (−0.00 pg/ml), and TNF-α (−0.11) significantly. TCJ supplementation did not also improve muscle soreness significantly, which was measured by VAS score (−2.81 mm). We found significant heterogeneity pertaining to IL-6 (*I*^2^ = 62.2%) and MVIC (*I*^2^ = 62.3%); therefore, we decided to perform subgroup analysis based on the type of drink, country, study design, gender, BMI, company, and type of exercise to discern the potential source(s) of heterogeneity. The sources of heterogeneity within our analysis merit a more comprehensive examination to understand why these factors might influence the outcomes.

In the context of IL-6, numerous variables have been identified as significant contributors to heterogeneity. Significantly, the variances in the results were influenced by factors such as the specific beverage type, the country in which the trials were done, and the study methodologies employed. It is essential to investigate the underlying causes behind the influence of these variables on the observed outcomes.

The potential influence of the type of drink on heterogeneity may arise from changes in antioxidant concentration, polyphenol composition, or other constituents between different types of TC products. Likewise, the geographical location where the research was conducted can bring diversity due to regional eating patterns, genetic variables, or other contextual impacts. The selection of a research design can have an influence on the outcomes, as it has the potential to affect the management of confounding variables and the accuracy of the measurements.

Surprisingly, we found that no significant heterogeneity was observed in the case of TC concentrate supplements, studies conducted in Australia, and studies with a crossover design. This implies that these particular components might have reduced the causes of heterogeneity, but it is essential to further investigate the underlying reasons behind this phenomenon.

Furthermore, it was revealed that the BMI and the particular type of exercise partly explained the observed heterogeneity in the IL-6 outcomes. It is worth exploring the mechanisms through which these individual characteristics interact with TCJ supplementation and influence IL-6 response.

Regarding MVIC, country was the most important source of heterogeneity and no heterogeneity was seen in studies that were conducted in Australia; however, type of drink, study design, gender, BMI, company, and type of exercise also partly explained the heterogeneity.

Our included studies used a variety of daily doses, from 60 to 1200 ml/day of TCJ, but after dose–response analysis for IL-6 and IL-8, we found that there was no significant difference between low dose (60 ml/day) and high dose (480 ml/day). Given the distinct findings around the optimal TCJ dose for enhancing MVIC, as well as the ambiguous effects on other biomarkers such as CK, further detailed analysis within both the Results and Discussion sections is warranted. This will highlight the nuances of TCJ supplementation, particularly in how different doses influence diverse outcomes. Since different types of TCJ have different content of anthocyanin, we decided to perform another dose–response analysis between the anthocyanin content of different TCJ and the final effect size, to investigate if the anthocyanin content of the juice is responsible for the improvement in MVIC. Results of this dose–response analysis indicated that there was no significant difference between lower (80 mg) and higher (548 mg) contents of anthocyanin; therefore, other compounds such as polyphenols and anti-oxidants might be responsible for such an effect, which were not reported by our included studies.

Unfortunately, no prior study investigated the safety and maximum dose of TCJ supplementation; however, results of prior studies indicated that 1200 ml/day was totally safe and did not cause significant side effects^[36]^.

We can conclude that TCJ supplementation is not a good strategy to reduce muscle damage, muscle soreness, and inflammation; however, it may be effective in muscle function improvement. To the best of our knowledge, no previous study investigated the effect of TCJ supplementation on EIMD in a dose–response manner; therefore, the present study sought to assess the effect of TCJ supplementation on different aspects of EIMD in a more comprehensive way in an athletic population that did not investigate before. In addition, Gao and Chilibeck demonstrated the ergogenic benefits of TC concentrate in endurance performance^[27]^. While their meta-analysis focuses on performance, our study adds insight into TCJ’s role in mitigating EIMD. Together, these findings highlight its diverse applications in athletic recovery and performance.

### Limitations

This study has several limitations. The low number of female participants (*N* = 46 vs. 166 males) limits generalizability, and subjective tools like the VAS may lack precision. Most studies had short durations (typically 8 days) and small sample sizes, restricting the detection of subtle effects. Few studies evaluated countermovement jump, pressure pain threshold, range of motion, or lipid hydroperoxides, leaving these markers underexplored. The dose–response analysis showed that 260 ml/day of TCJ yielded maximum MVIC benefits, with no effects at lower doses and no added benefits at higher doses. However, no clear link to anthocyanin content was found, suggesting other unmeasured components may play a role. High heterogeneity across indicators, likely due to differences in designs and participant characteristics, underscores the need for standardized protocols and improved reporting. Lastly, none of the studies reported on TCJ’s side effects or toxicity, highlighting the need for future research to assess its safety.

### Optimal supplementation dose

Results of the present systematic review and meta-analysis suggest that supplementation with 260 ml/day of TCJ can significantly improve MVIC levels.

## Conclusion

The present study provides evidence that TCJ supplementation may significantly improve factors involved in the process of EIMD including MVIC and some inflammatory biomarkers such as IL-6 and IL-8. However, we did not have such an effect on muscle damage, muscle soreness, and other inflammatory biomarkers such as CRP, TNF-α, and IL-1β. Therefore, TCJ supplementation appears to represent a safe and effective dietary strategy to improve muscle function in athletic population. Results of dose–response analysis of the present study indicate that 260 ml/day of TCJ is the optimal dose to improve MVIC levels. Further well-designed high-quality studies are needed to firmly establish the clinical efficacy of this plant. Due to the short duration of most studies, as well as a comparable lack of female participants, it is suggested that further well-designed studies with longer duration of intervention focusing on female athletes be conducted. It is also suggested that the effect of TCJ supplementation on athletic performance and recovery be examined.

## Data Availability

The data that support the findings of this study are available from the corresponding author upon reasonable request.

## Research registration unique identifying number (UIN)

Not applicable. This is a systematic review and meta-analysis rather than a primary research study requiring registration.
